# Biomedical named entity recognition with the combined feature attention and fully-shared multi-task learning

**DOI:** 10.1186/s12859-022-04994-3

**Published:** 2022-11-03

**Authors:** Zhiyu Zhang, Arbee L. P. Chen

**Affiliations:** 1 Department of Computer Science, National Tsing Hua University, Hsinchu, Taiwan; 2 Department of Computer Science and Information Engineering, Asia University, Taichung, Taiwan

**Keywords:** Named entity recognition, Biomedical text mining, Syntactic information, Multi-task learning, Attention

## Abstract

**Background:**

Biomedical named entity recognition (BioNER) is a basic and important task for biomedical text mining with the purpose of automatically recognizing and classifying biomedical entities. The performance of BioNER systems directly impacts downstream applications. Recently, deep neural networks, especially pre-trained language models, have made great progress for BioNER. However, because of the lack of high-quality and large-scale annotated data and relevant external knowledge, the capability of the BioNER system remains limited.

**Results:**

In this paper, we propose a novel fully-shared multi-task learning model based on the pre-trained language model in biomedical domain, namely BioBERT, with a new attention module to integrate the auto-processed syntactic information for the BioNER task. We have conducted numerous experiments on seven benchmark BioNER datasets. The proposed best multi-task model obtains F1 score improvements of 1.03% on BC2GM, 0.91% on NCBI-disease, 0.81% on Linnaeus, 1.26% on JNLPBA, 0.82% on BC5CDR-Chemical, 0.87% on BC5CDR-Disease, and 1.10% on Species-800 compared to the single-task BioBERT model.

**Conclusion:**

The results demonstrate our model outperforms previous studies on all datasets. Further analysis and case studies are also provided to prove the importance of the proposed attention module and fully-shared multi-task learning method used in our model.

## Background

With the rapid development of biomedical research, the number of biomedical documents increases with the explosive exponential growth, which has made it difficult for biomedical scholars to keep pace with the cutting-edge research. There is an increasing need of effective natural language processing (NLP) tools to help retrieve, organize, and manage the massive biomedical data and information. Biomedical named entity recognition (BioNER) is a primary first step in any biomedical literature mining task, which aims to detect the boundary of biomedical entities and predict their entity types, such as diseases, genes, species, chemical, etc. The performance of BioNER systems directly impacts downstream applications, such as biomedical relation extraction [1, 2], drug-drug interaction task [3, 4] and knowledge base construction [5, 6].

**Fig. 1:**
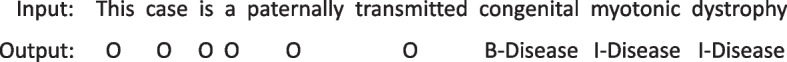
An example sentence for the input and output in BioNER

A BioNER task is typically considered as a sequence labeling task, which aims to assign the best label sequence for a given input sentence. A common tagging method is the BIO format [7], which denotes whether each token is at the **B**eginning of an entity, **I**nside, or **O**utside an entity. This method is capable of distinguishing consecutive entities and can be used easily in an end-to-end model, which inputs each token and produces BIO tags in the final layer. An example sentence annotated using the BIO format can be found in Fig. 1, where “congenital myotonic dystrophy” is the entity detected and “disease” is the entity type classified.

Traditional methods for the BioNER task usually used dictionary-based or rule-based approaches [8, 9]. These methods heavily relied on biomedical experts to establish dictionaries or rules, which takes a lot of manual labor and is time consuming. As the amount of data increases, more researchers tried to use machine learning approaches to deal with the BioNER task, such as Support vector machine (SVM) [10, 11] or Conditional random field (CRF) [7, 12, 13]. However, the conventional machine learning approaches need plenty of handcrafted features extracted from raw data, and the performance is limited. The rapid development of deep learning provides an easier way to overcome these problems. Crichton et al. [14] used the word context as the input based on the convolutional neural network (CNN) and Habibi et al. [15] proposed the bidirectional LSTM (BiLSTM) model combined with a CRF layer. More recently, pre-trained language models like BERT [16], XLNet [17], and Roberta [18] achieved great success on a lot of NLP tasks. Lee et al. [19] introduced a domain-specific language model, named *BioBERT*, which is pre-trained on the large-scale biomedical corpora. BioBERT largely outperformed previous methods in several biomedical text mining tasks including BioNER task. Considering the powerful performance of BioBERT, we propose to use it as the encoder of our model to obtain high-quality semantic representations.

**Fig. 2:**
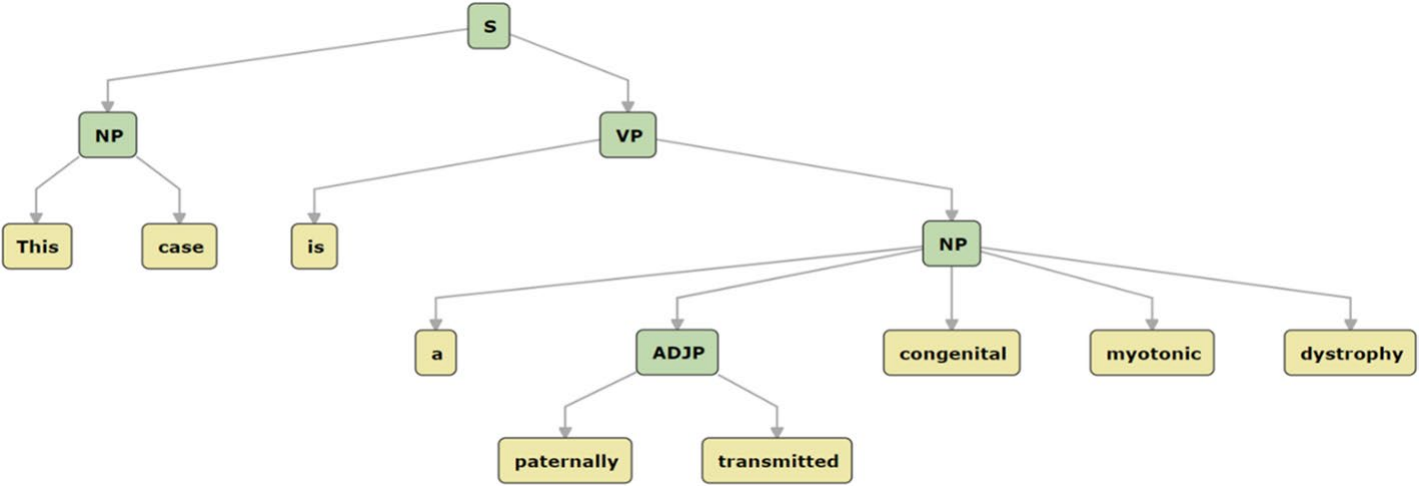
A constituency parse tree example

In addition, we assume that combining the syntactic information, e.g., part-of-speech (POS) labels, syntactic constituents, and dependency relations with the pre-trained BioBERT can help recognize biomedical named entities. Specifically, sentences in biomedical texts are usually formal, well-structured and contain a lot of specialized terms, in which syntactic information can present grammatical structure for sentences and provide helpful cues for understanding the relationship between words. For example, Fig. 2 shows the constituency parse tree automatically produced by the NLP toolkit, where the disease entity is “congenital myotonic dystrophy.” The range of the noun phrase in this tree instead of the adjective phrase can be a good hint for BioNER. The other advantage of syntactic information is that it can be automatically generated by off-the-shelf NLP toolkits rather than manually constructed, which makes it easier to use in this task. Previous studies [20–24] suggest that the syntactic information has a certain ability to help the BioNER task. These studies normally concatenated the embeddings of the syntactic features with the word embeddings directly, which hurts the model performance because of the error-prone and noisy syntactic information processed from NLP toolkits. Accordingly, Tian et al. [25] proposed a novel model instead of directly concatenating to incorporate the syntactic information into the BioBERT encoder and achieved the best results in several BioNER datasets. They used the key-value memory network (KVMN) [26], a new deep neural method learning from pairwise information, to weight the syntactic features. However, the output of the KVMN mainly relies on the value embeddings. The key embeddings are only used for providing weights to values. To solve this problem, Tian et al. [27] proposed a new attention mechanism, named *two-way attention*, to integrate syntactic information for the encoder. The two-way attention can make full use of syntactic features, rather than using one feature (key embeddings) to weight the other (value embeddings) as in KVMN. Although this method achieved good performance in another task, named “the joint Chinese word segmentation (CWS) and part-of-speech (POS) tagging task,” it still has some shortcomings. One is that the two-way attention mechanism employed two separate attention parts, therefore it may lose some information between the two parts. Another is that the embeddings of syntactic information are only randomly initialized, which lacks a strong semantic representation ability and may cause the out-of-vocabulary problem. Consequently, we propose a novel attention mechanism to tackle these problems.

An effective deep learning model requires huge amounts of data. However, the dataset in the biomedical domain is more likely to become unavailable due to the limitations of privacy and specialization. To deal with the above problems, multi-task learning (MTL) has been introduced by previous studies [14, 28, 29, 29–34] and achieved great success in the BioNER task. The basic method of MTL is that multiple annotated datasets are trained at the same time to improve the performance on a single dataset. The datasets are all used in the BioNER task with a similar format, which may have different entity types and may be created by different researchers. Different datasets in the similar domain may contain useful common information like lexical semantics and grammatical expression. The multi-task model can therefore share this information across different datasets in the training step. In general, previous MTL models have adopted the strategy that the model shares certain parts of the model parameters for different datasets and leaves the rest separated for specific tasks. For example, Crichton et al. [14] proposed an MTL model by sharing parameters in encoder layers and convolution layers, and trained separately in decoder layers for each dataset. Wang et al. [28] proposed a BiLSTM-CRF model with an additional character layer. An MTL model was trained by sharing parameters of the character-level and word-level LSTMs and adjusting parameters of the CRF layer independently for different datasets. Chai et al. [34] trained an MTL model by sharing parameters in underlying layers of the XLNet and trained separately the upper layers of the XLNet and the decoder CRF. This MTL method leads to the limited ability of sharing information across different datasets and causes the model to rely too heavily on the task-specific layer. Besides, the multi-task model parameters will increase with the number of datasets because of additional task-specific layers. Huang et al. [35] proposed a transfer learning model by sharing all parameters to integrate multiple cross-domain datasets to achieve good results in Chinese Word Segmentation tasks. Inspired by this work, we propose a straightforward and effective multi-task learning model that shares all parameters across different datasets. The benefit is that we do not need any task-specific models to fit different datasets, and the method can be directly applied in the single-task model without manual adjustments. It can control the rapid growth of the total parameters on the MTL model and improve the performances on several BioNER datasets.

In this paper, we propose a novel fully-shared multi-task learning model based on the pre-trained BioBERT with a new attention module to integrate the auto-processed syntactic information for the BioNER task. The proposed framework contains two parts: One is the single-task method which is only trained on each single BioNER benchmark dataset, and the other is the multi-task method trained across all datasets together. Specifically, our single-task model uses a new proposed attention mechanism, named *Combined Feature Attention* (CFA), to integrate the syntactic information into BioBERT encoder for improving the performance. We employ the open source NLP toolkit to parse the input sentence and extract several types of syntactic information. Then, we use the proposed attention module to weight each token and its corresponding syntactic features, where syntactic features are combined with the hidden embeddings derived from BioBERT and syntactic labels obtained from the toolkit. Finally, the attention vectors are concatenated with the output of the BioBERT and used to guide the tagging process for the decoder. In this way, the single-task method takes advantage of the pre-trained BioBERT and syntactic information, and outperforms other single-task models in the BioNER task. Moreover, we introduce a straightforward and effective multi-task learning method which shares all model parameters to incorporate multiple datasets into one model. The fully-shared MTL method is a basic but effective way to learn the commonality among different datasets and can be applied easily to many single-task neural network models. To summarize, the main contributions of this paper are as follows:

- We propose a new attention mechanism, named CFA, to make good use of the pre-trained BioBERT and the syntactic information in the single-task model. Our single-task model substantially outperforms the baseline BioBERT model and other models using the syntactic information because of our better syntactic feature extraction and combination ability.
- We introduce a straightforward and effective multi-task learning method which shares all parameters without task-specific layers for different datasets. The fully-shared MTL method discriminatively exploits the implicit information across different datasets and significantly improves BioNER compared with the single-task model.
- The experiment results on seven benchmark BioNER datasets show our fully-shared MTL model with CFA outperforms others on all datasets, which proves the effectiveness of the proposed method. Analyses and case studies show all components of our proposed model are necessary for achieving high performance.

## Methods

Following the previous approaches, we treat BioNER as a sequence labeling task. Given the input biomedical sentence of n words $\text {X}=[{{x}_{1}},{{x}_{2}},\ldots ,{{x}_{i}},\ldots ,{{x}_{n}}]$, the output is a sequence of named entity labels $\text {Y}=[{{y}_{1}},{{y}_{2}},\ldots ,{{y}_{i}},\ldots ,{{y}_{n}}]$, where ${{x}_{i}}$ is the i-th word in the sentence, and ${{y}_{i}}$ is the i-th predicted label. For each ${{x}_{i}}$, the goal is to predict the corresponding label ${{y}_{i}}$ ‘B’, ‘I’, ‘O’, where ‘B’ indicates the word ${{x}_{i}}$ is the beginning of a biomedical entity, ‘I’ denotes ${{x}_{i}}$ is inside an biomedical entity, and ‘O’ denotes ${{x}_{i}}$ is outside an entity, i.e. ${{x}_{i}}$ is not a part of an biomedical entity.

The proposed framework contains two parts: One is the single-task model which is only trained on each single BioNER dataset, and the other is the multi-task model trained across all datasets together. In this section, we respectively explain details of the proposed single-task model and multi-task model.

**Fig. 3:**
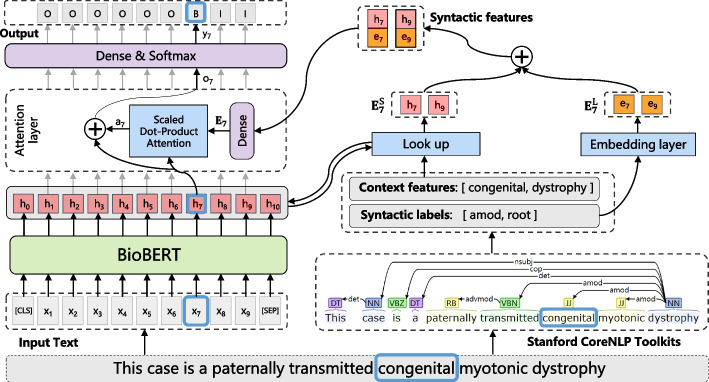
The architecture of the proposed single-task model for BioNER (the context features and syntactic labels of the 7th word “congenital” in the example sentence are extracted from the results processed by the NLP toolkit)

### Single-task model (STM)

The overall architecture of our single-task model is detailed in Fig. 3. The left part describes the backbone of the proposed architecture for the BioNER sequence labeling paradigm, and the right part is the process of handling the syntactic information. We propose a novel attention module to integrate the syntactic information into the backbone of the model. In this section, the process about syntactic feature extraction is first introduced. Next, we describe how the proposed attention mechanism, namely combined feature attention (CFA), incorporates the syntactic features into BioBERT. Finally, we describe how the sequence labeling model works with the attention layer.

#### Syntactic feature extraction

**Fig. 4:**
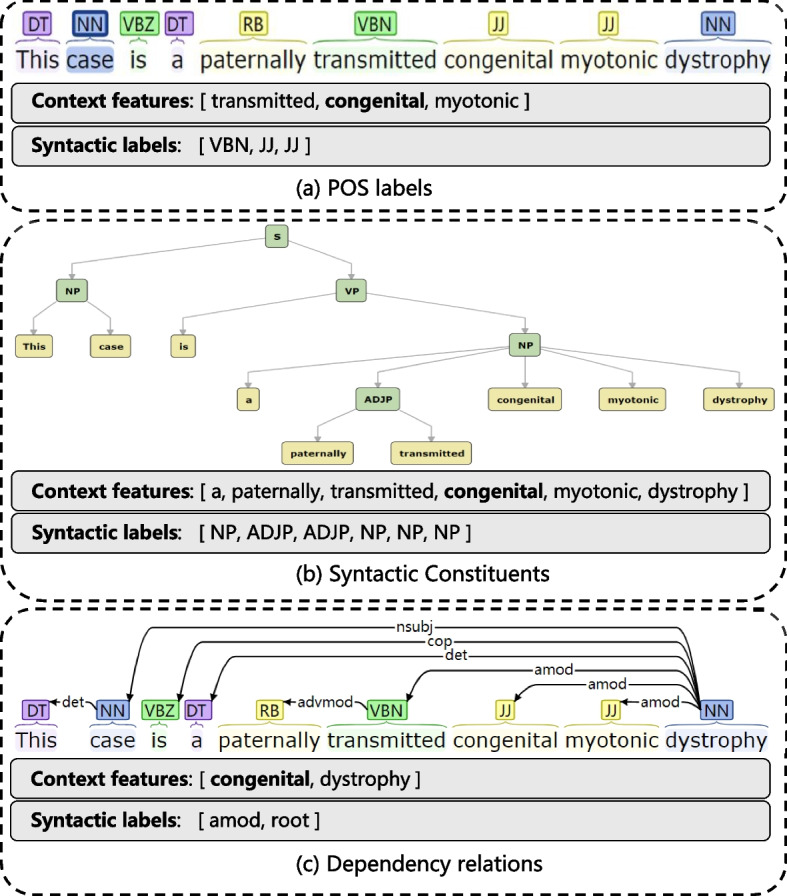
An example of syntactic feature extraction

Following previous studies [25, 27], we utilize three types of syntactic information: POS labels, syntactic constituents, and dependency relations. POS is a category of words with similar grammatical properties, such as nouns, verbs, adjectives, adverbs and so on. Syntactic constituent is a word or a group of words that functions as a single unit in a hierarchical structure, such as a noun phrase, or verb phrase. Dependency relations are the concept that words are connected to each other through some kind of directed links, such as nominal subject, copula, adjectival modifier and so on. To obtain the syntactic information, first, we run the open source NLP toolkit, e.g., Stanford CoreNLP Toolkit (SCT) [36] to get the results for the input sentence $\text {X}$. Then we extract the context features and their corresponding syntactic labels of each word ${{x}_{i}}$ in $\text {X}$ from the results. In Fig. 4, we show an example for the highlighted word “congenital” in the input sentence “This case is a paternally transmitted congenital myotonic dystrophy,” where three types of context feature and their corresponding syntactic labels are extracted. We elaborate each type of syntactic information below.

- *POS labels* Consider each word ${{x}_{i}}$ in sentence $\text {X}$, we employ a 1-word window to extract the neighboring words on both sides of ${{x}_{i}}$ as context features and their corresponding POS labels as syntactic labels. For example, in Fig. 4a, the word “congenital” is the currently processed word, then the context features are the word itself and its left and right neighboring words and syntactic labels are the corresponding POS labels of each context word obtained from the toolkit. The context features are [transmitted, congenital, myotonic], and the syntactic labels are [VBN, JJ, JJ].
- *Syntactic constituents* Given a word ${{x}_{i}}$ in $\text {X}$, we first find the leaf containing ${{x}_{i}}$ in the syntactic parse tree, and then search up from the leaf to find the first acceptable ancestor node whose label is in a pre-defined syntactic label list following “the CoNLL-2003 shared task” [37]. Then we select all the words under this node as context features and search first ancestor nodes of these words as their corresponding syntactic labels. In Fig. 4b, “NP” is the first acceptable ancestor node for the example word “congenital.” There are six words under this node and each word can find its ancestor node. The context features are [a, paternally, transmitted, congenital, myotonic, dystrophy] and the corresponding syntactic labels are [NP, ADJP, ADJP, NP, NP, NP].
- *Dependency relations* Dependency relations use directed acyclic graphs to depict the structure of a given sentence. The asymmetric relationship between two basic units has been called the dependency relation. One unit is the dominant element (called *governor*), and the other is the subordinate element (called *dependent*). For each word ${{x}_{i}}$ in $\text {X}$, we first select governor words and dependent words of ${{x}_{i}}$ from the dependency structure as shown in Fig. 4c. Then, we treat these governor words, dependent words and the word ${{x}_{i}}$ as context features and treat the dependency types of these words in the graph as syntactic labels. As is shown in Fig. 4c, the example word “congenital” has only one governor word “dystrophy” which is the root of the sentence and no dependent words. For comparison, the word “transmitted” has one dependent word “paternally” which is pointed from “transmitted.” The context features for the word “congenital” are [congenital, dystrophy] and the corresponding syntactic labels are [amod, root].

After these procedures, we can build the context feature sequence S and the syntactic label sequence L for each type of the syntactic information for each input sentence $\text {X}$. Formally, for each word ${{x}_{i}}$ in $\text {X}$, let ${\text {S}_{i}}=[{{s}_{i,1}},{{s}_{i,2}},\ldots ,{{s}_{i,j}},\ldots ,{{s}_{i,{m}_{i}}}]$ and ${\text {L}_{i}}=[{{l}_{i,1}},{{l}_{i,2}},\ldots ,{{l}_{i,j}},\ldots ,{{l}_{i,{m}_{i}}}]$ be the sub sequence of S and L, respectively. Here, ${{s}_{i,j}}$ denotes a context word extracted by the rules we define, ${{l}_{i,j}}$ denotes the corresponding syntactic label for ${{s}_{i,j}}$, and ${m}_{i}$ denotes the length of ${\text {S}_{i}}$ and ${\text {L}_{i}}$. For example, in Fig. 4a, we focus on the 7th word “congenital,” ${{s}_{7,1}}$ = “transmitted,” ${{l}_{7,1}}$ = “VBN,” and ${m}_{7}$ =3. It’s worth noting that we obtain different S’s and L’s for three types of syntactic information, and our model utilizes each type of syntactic information separately.

#### Combined feature attention

Inspired by Tian et al. [27], we use the attention method to incorporate the syntactic features into the BioBERT model. We first feed the input sentence X into the encoder pre-trained BioBERT to get the hidden vector sequence:

1 $$\begin{aligned} {{\mathbf {H}}}=[\mathbf {h}_{1},\mathbf {h}_{2},\ldots ,\mathbf {h}_{i},\ldots ,\mathbf {h}_{n}] \end{aligned}$$

where $\mathbf {h}_{i}\in {{\mathbb {R}}^{{d}_{1}}}$ is the hidden vector of the i-th word ${{x}_{i}}$ and ${{d}_{1}}$ is the hidden dimension of the encoder. Second, we change the context feature sequence ${{S}_{i}}$ and syntactic label sequence ${{L}_{i}}$ to embedding matrices respectively for each word ${{x}_{i}}$. Because the words in ${{S}_{i}}$ are also included in the input sentence X, we leverage the hidden vector sequence H to embed ${{S}_{i}}$. Different from previous methods [25, 27] that use randomly initialized embeddings or pre-trained embeddings, the embeddings in our method have more abundant semantic representations and can avoid the OOV problem due to the powerful function and good performance of BioBERT. Specifically, a context word ${{s}_{i,j}}$ in ${{S}_{i}}$ is probably the k-th word in the sentence X, so we use $\mathbf {h}_{k}$ to directly represent the embedding of ${{s}_{i,j}}$, where 1$\le$k$\le$n . In this way, we can obtain the embedding of each word in ${{S}_{i}}$:

2 $$\begin{aligned} \mathbf {{E}_{i}^\text {S}}=[\mathbf {e}_{{i,1}}^\text {S},\mathbf {e}_{{i,2}}^\text {S},\ldots ,\mathbf {e}_{{i,j}}^\text {S},\ldots , \mathbf {e}_{i,{m}_{i}}^\text {S}] \end{aligned}$$

where the context feature embedding matrix $\mathbf {{E}_{i}^\text {S}}\in {{\mathbb {R}}^{{d}_{1}\times {m}_{i}}}$ and $\mathbf {e}_{{i,j}}^\text {S}=\mathbf {h}_{k}$. As is shown in Fig. 3, the context features for the word “congenital” in the type of dependency relations are [congenital, dystrophy], where “congenital” and “dystrophy” are the 7th and 9th word in the input sentence, so $\mathbf {{E}_{i}^\text {S}}=[\mathbf {e}_{{i,1}}^\text {S},\mathbf {e}_{{i,2}}^\text {S}]=[\mathbf {h}_{7},\mathbf {h}_{9}]$. As for ${{L}_{i}}$, we adopt the common approach of randomly initializing the embeddings and training with the model:

3 $$\begin{aligned} \mathbf {{E}_{i}^\text {L}}=[\mathbf {e}_{{i,1}}^\text {L},\mathbf {e}_{{i,2}}^\text {L},\ldots ,\mathbf {e}_{{i,j}}^\text {L},\ldots , \mathbf {e}_{i,{m}_{i}}^\text {L}] \end{aligned}$$

where the syntactic label embedding matrix is $\mathbf {{E}_{i}^\text {L}}\in {{\mathbb {R}}^{{d}_{2}\times {m}_{i}}}$ and ${d}_{2}$ is the artificially set dimension of the initial embeddings. Then, we concatenate $\mathbf {{E}_{i}^\text {S}}$ and $\mathbf {{E}_{i}^\text {L}}$ to obtain syntactic feature embedding matrix for each input word ${{x}_{i}}$ and align the dimension of $\mathbf {e}_{{i,j}}$ and $\mathbf {h}_{k}$ by a fully connected layer:

4 $$\begin{aligned} \mathbf {e}_{{i,j}}= & {} \mathbf {{W}_{e}}\cdot (\mathbf {e}_{{i,j}}^\text {S}\oplus \mathbf {e}_{{i,j}}^\text {L})+\mathbf {{b}_{e}} \end{aligned}$$

5 $$\begin{aligned} \mathbf {{E}_{i}}= & {} [\mathbf {e}_{{i,1}},\mathbf {e}_{{i,2}},\ldots ,\mathbf {e}_{{i,j}},\ldots ,\mathbf {e}_{i,{m}_{i}}] \end{aligned}$$

where $\mathbf {{E}_{i}}\in {{\mathbb {R}}^{{d}_{1}\times {m}_{i}}}$ is the syntactic feature embedding matrix for ${{x}_{i}}$, $\mathbf {{W}_{e}}\in {{\mathbb {R}}^{{d}_{1}\times ({d}_{1}+{d}_{2})}}$ is the weight matrix and $\mathbf {{b}_{e}}\in {{\mathbb {R}}^{{d}_{1}\times {m}_{i}}}$ is the bias vector.

Finally, we apply the Scaled Dot-Product Attention [38], an effective attention mechanism used in many NLP tasks, with the representations of the word ${{x}_{i}}$ and its syntactic feature to get attention vectors. It can be formulated as:

6 $$\begin{aligned} {{a}_{i,j}}=\text {softmax}\left( \frac{{(\mathbf {h}_{i})^{\mathsf {T}}}\cdot {\mathbf {e}_{{i,j}}}}{\sqrt{{d}_{1}}}\right) \end{aligned}$$

7 $$\begin{aligned} \mathbf {{a}_{i}}=\sum \limits _{j=1}^{{m}_{i}}{{{a}_{i,j}}\cdot {\mathbf {e}_{{i,j}}}} \end{aligned}$$

where ${a}_{i,j}\in {{\mathbb {R}}^{1}}$ is the attention weight for each syntactic feature $\mathbf {e}_{{i,j}}$ in $\mathbf {{E}_{i}}$, $\mathbf {{h}_{i}}$ is the hidden vector of ${{x}_{i}}$, $\mathbf {{a}_{i}}\in {{\mathbb {R}}^{{d}_{1}}}$ is the weighted vector for all syntactic features in $\mathbf {{E}_{i}}$ and $\sum$ denotes an element-wise sum operation. After that, we concatenate $\mathbf {{a}_{i}}$ and $\mathbf {{h}_{i}}$ to get the output vector $\mathbf {{o}_{i}}$ for each word ${{x}_{i}}$ in sentence X, which can be expressed by $\mathbf {{o}_{i}}=\mathbf {{a}_{i}}\oplus {\mathbf {{h}_{i}}}$.

In this way, the proposed attention module can learn the weights of the corresponding syntactic features for the input sentence. Since the attention module uses a special embedding method which combines the information of context features and syntactic labels, we name it combined feature attention (CFA).

#### Sequence tagging network

Once the output vector $\mathbf {{o}_{i}}$ is obtained from the CFA module, we feed it into a fully-connected layer followed by a softmax layer. For each word ${{x}_{i}}$ in X, the tagging probability distribution $\mathbf {\hat{y}}$ can be formulated as follows:

8 $$\begin{aligned} \mathbf {\hat{y}}=[{{\hat{y}}_{1}},{{\hat{y}}_{2}},{{\hat{y}}_{3}}]=\text {softmax}[\mathbf {W}\cdot \mathbf {{o}_{i}}+\mathbf {b}] \end{aligned}$$

where $[{{\hat{y}}_{1}},{{\hat{y}}_{2}},{{\hat{y}}_{3}}]$ denote the probability of each type of BioNER labels, i.e., “B,” “I” and “O,” $\mathbf {W}$ and $\mathbf {b}$ are trainable parameters. We can also use the CRF layer instead of the softmax layer in our model, but from our test experiments it did not achieve significant improvement and took longer time in training steps. The loss function is cross-entropy.

### Multi-task model (MTM)

**Fig. 5:**
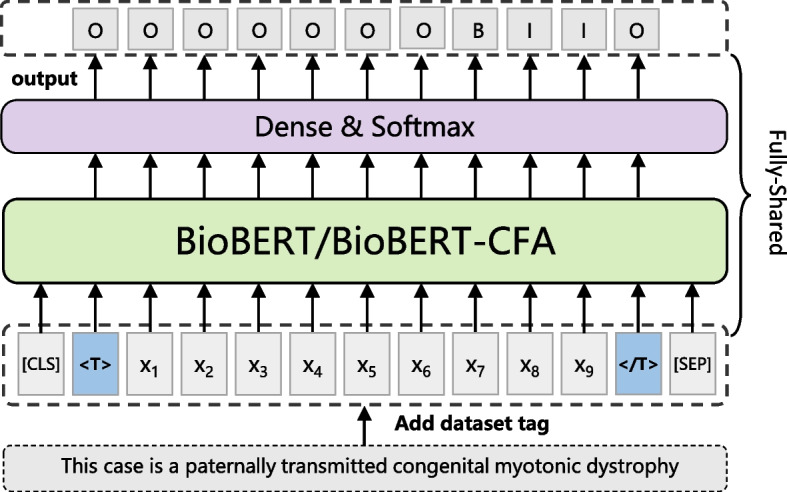
The architecture of the proposed multi-task learning model for BioNER

Recently, multi-task learning (MTL) has been successfully applied to solve the problem of limited availability of annotated data in the BioNER tasks. Most previous MTL models for the BioNER task use multiple datasets simultaneously to train a model, in which some parameters of the model are shared for different datasets and the others are separated and task-specific. This leads to the limited ability of sharing information across different datasets and the explosive growth of the total parameters with the increase of datasets. We propose a straightforward and effective MTL method to attach a pair of tag identifiers for each input word sequence, “$<tag>$” and “$</tag>$,” at the beginning and end of the sequence respectively, where “tag” denotes the name of the dataset containing the input sentence. As is shown in Fig. 5, if the input sentence X belongs to “T” dataset, we add the tag “$<T>$” before the first word ${x}_{1}$ and add the tag “$</T>$” after the last word ${x}_{9}$.

In the training step, we input sentences from all different datasets together with tag identifiers into the model. These tag identifiers distinguish the origin of each sentence to affect the hidden representations of each word in the sentence. It is similar to directly telling the model which dataset the input sentence belongs to, and allowing the model to learn the differences and commonalities between datasets. Since we only change the input sentence before encoding without the model architecture modification, the proposed fully-shared MTL method can share all parameters in the training step to integrate different datasets and train the model without any task-specific layers. In addition, BioNER datasets include various biomedical entity types, such as gene, disease, and species. There are multiple datasets for each type. Although the datasets under different types are quite different, we assume that the cross-type information of the biomedical domain can improve the performance of the multi-task model. Therefore, we train the model on the datasets under multiple entity types at the same time. Moreover, for the tag identifier “$<tag>$,” we can use the name of the dataset or entity type containing the input sentence. Since the datasets under the same type are still different due to different constructors and annotation rules, we decide to use the name of the dataset as the tag. If we use the name of the entity type, the differences between datasets will not be captured.

In the inference step, we predict specific test sets by adding the corresponding dataset tag to the input sentence. If you want to recognize entities for a biomedical sentence, you need to select an appropriate dataset tag used in the training step according to your purpose. Different choices will lead to different results. For example, when you want to detect disease entities, you should choose any of the dataset belonging to disease type as the tag identifier.

## Results

In this section, we first describe several BioNER benchmark datasets used in our experiments. Then we introduce the experimental setup and implementation details. Next, we present the results of different experiments for the proposed single-task model and multi-task model, respectively.

### Datasets

We make experiments on seven BioNER benchmark datasets^1^ which are publicly available and widely used in previous studies. We utilize the same splitting strategy on training, validation and testing sets according to Lee et al. [19] for each dataset. Since these datasets include various biomedical entity types, we divide them into four categories: gene/protein, disease, species and chemical. Table 1 gives some details of these datasets including the number of sentences, sentence length, entity type and entity count, where the sentence length represents the average length of the sentences in the dataset, and entity count represents the total number of entities mentioned in the dataset. More details about these datasets can be found in [14].

**Table 1:** The statistics of the datasets used in our experiments

Dataset	Number of sentences	Sentence length	Entity type	Entity count
BC2GM	20,000	28.5	Gene/protein	24,583
JNLPBA	24,806	29.7		35,336
BC5CDR-disease	13,938	26.0	Disease	12,852
NCBI-disease	6881	26.1		6881
Linnaeus	23,155	23.1	Species	4263
Species-800	8130	25.9		3651
BC5CDR-chemical	13,938	26.0	Chemical	15,935

### Experiment setup

Our experiments are divided into two parts. We train the proposed single-task model (STM), named *BioBERT-CFA*, and some other comparative STMs for each of the datasets. Then we train the multi-task model (MTM) with all datasets jointly by using the proposed MTL method based on the vanilla BioBERT model and the proposed BioBERT-CFA model.

For the experiments of STM, we use “Stanford CoreNLP Toolkits” (SCT) [36] , a well-known open source toolkit which is widely used in many NLP studies, to process ach input sentence and obtain parts-of-speech, constituency, and dependency parsing results as the syntactic information. We use each type of syntactic information separately in the CFA module. For the encoder, we use the base v1.1 version of BioBERT^2^ and keep the default hyper-parameter followed by Lee et al. [19], which consists of 12 transformer layers with 768 hidden vector dimensions. The parameters in the BioBERT encoder are fine-tuned with the model training. The embeddings of context features are derived from BioBERT and the embeddings of syntactic labels are randomly initialized in the CFA module. For the experiments of MTM, we combine all datasets as a total dataset for training. Then we change each input sentence respectively in the total dataset by using the proposed MTL method and feed it into STM to train a multi-task model. In testing, we evaluate the results of each dataset separately for each multi-task model.

We implement all experiments on a NVIDIA Tesla V100 GPU using PyTorch library^3^. We employ Adam [39] as the optimizer with the learning rate of 5e-5 and train each model with a batch size of 64 and maximum sequence length of 128 for 30 epochs. For the evaluation metrics of BioNER, we use macro-averaged F1 scores computed by the widely used *seqeval*^4^ script in all experiments.

### Single-task model results

For comparison, we adopt the following three single-task models for the BioNER task as the baselines: the first one is the vanilla BioBERT model proposed by Lee et al. [19], which achieved good performance in many biomedical tasks. The second one is named *BioKMNE* [25] based on a key-value memory network (KVMN) [26] to integrate the syntactic feature with BioBERT and it outperformed the vanilla BioBERT model in their experiments. Besides, we implement a novel attention mechanism, named *two-way attention* (TWA) proposed for other tasks by Tian et al. [27], instead of the KVMN module in the BioKMNER model to incorporate the syntactic information for the BioNER task. We name this model *BioBERT-TWA* and assume that BioBERT-TWA can outperform BioKMNER. The BioKMNER model, BioBERT-TWA model and the proposed single-task model BioBERT-MFA use the same three types of syntactic information: POS labels (POS), syntactic constituents (Syn), and dependency relations (Dep) and employ the same NLP toolkit SCT to get the syntactic information.

Table 2 shows the overall performance of our model BioBERT-CFA compared with the three baseline models on the seven benchmark datasets, where *BioBERT (ours)* denotes our reproduced results of BioBERT, *BioBERT-TWA (ours)* denotes our reproduced results of the two-way attention method with the BioBERT encoder, and *BioBERT-CFA (ours)* denotes the results of our proposed single-task model. Bold indicates the highest score among all models. There are several observations for these results.

**Table 2:** Performance of different single-task models

Model	BC2GM	JNLPBA	BC5CDR-Disease	NCBI-disease	Linnaeus	Species-800	BC5CDR-Chemical
BioBERT	84.72	77.49	87.15	89.71	88.24	74.06	93.47
BioBERT (ours)	84.62	77.41	86.89	89.18	88.06	74.88	93.44
BioKMNER							
POS	84.74	77.06	–	89.47	88.44	75.45	93.73
Syn	84.76	77.17	–	89.27	88.68	75.65	93.74
Dep	84.65	77.32	–	89.24	88.57	75.81	93.78
BioBERT-TWA (ours)							
POS	84.83	77.81	86.91	89.41	88.17	75.03	93.52
Syn	84.96	78.02	87.03	89.36	88.42	75.24	93.68
Dep	84.85	78.23	87.12	89.52	88.36	75.14	93.81
BioBERT-CFA (ours)							
POS	85.06	78.21	87.45	**89.98**	88.38	75.62	93.89
Syn	**85.36**	78.36	87.49	89.91	**88.75**	**75.83**	94.05
Dep	85.28	**78.47**	**87.56**	89.94	88.66	75.64	**94.09**

Firstly, compared with the vanilla BioBERT model without using any syntactic information, all models incorporating syntactic information achieve better results among most datasets. It demonstrates the effectiveness of using syntactic information to help recognize biomedical named entities.

Secondly, comparing BioKMNER and BioBERT-TWA, we find that BioBERT-TWA yields better performance in most cases. For instance, on the BC2GM dataset, BioBERT-TWA (Syn) achieves the F1 score of 84.96%, while KMNER obtains a lower F1 score of 84.76%. This phenomenon that the performance of KVMN is not as good as TWA is consistent with the results in Tian et al. [27], which may be due to the reason that the method of computing weights in KVMN is inaccurate compared to TWA.

Thirdly, the proposed single-task model BioBERT-CFA achieves the best performance on all benchmark datasets and provides a significant enhancement to the baselines by incorporating the syntactic information. For example, BioBERT-CFA achieves improvements of 1.06%, 0.95% and 0.80% F1 scores for the JNLPBA, Species-800 and NCBI-disease datasets respectively compared with BioBERT, which confirms the effectiveness and universality of the proposed CFA module. Comparing with BioBERT-TWA, the BioBERT-CFA model uses a novel attention mechanism and embedding method, and provides outstanding performance.

Among different types of syntactic information, in most cases, syntactic constituents (Syn) and dependency relations (Dep) in our experiments work better than part of speech tags (POS). For example, the BioBERT-CFA model achieves 85.36% and 85.28% F1 scores on the BC2GM dataset when it uses Syn and Dep, respectively, while 85.06% is achieved when it uses POS labels. The same phenomenon can be found in BioKMNER and BioBERT-TWA models. This is partly because the syntactic constituents and dependency relations provide more cues of the relationship between words, while the POS labels focus more on attributes of the word itself.

### Multi-task model results

We train the multi-task model (MTM) with all aforementioned datasets together by using the fully-shared multi-task learning method based on the vanilla BioBERT model and the proposed BioBERT-CFA model, named *BioBERT-MTM* and *BioBERT-CFA-MTM*, respectively. In BioBERT-CFA-MTM, we use dependency relations (Dep) as syntactic information because of its good performance. The BioBERT-STM is our baseline model which denotes the single-task BioBERT model trained by a single dataset separately. For comparison, we design a BioBERT-DM model where we train BioBERT on the whole dataset which directly mixes all datasets without our MTL method. As shown in Table 3, the BioBERT-DM model greatly hurt the performance on all datasets because different datasets have different entity types and annotation rules, and this model has no ability to distinguish between different datasets. In contrast, the BioBERT-MTM yields quite stable improvements no matter what dataset we test compared with the baseline, which confirms that the model trained jointly with different datasets by using the fully-shared MTL method would achieve better performance than training it by a single dataset. The fully-shared MTL method can learn useful information from different datasets. The F1 scores of BioBERT-MTM on BC5CDR-Chemical dataset only increase by 0.12%, which is because that BC5CDR-Chemical is the only one of chemical types of those seven datasets. Additionally, we find that the final model BioBERT-CFA-MTM enhances performance remarkably on all datasets, which again shows the effectiveness of the proposed CFA module and MTL method.

**Table 3:** Results of different multi-task models

Model	BC2GM	JNLPBA	BC5CDR-Disease	NCBI-disease	Linnaeus	Species-800	BC5CDR-Chemical
BioBERT-STM	84.62	77.41	86.89	89.18	88.06	74.88	93.44
BioBERT-DM	77.63	72.27	77.98	81.29	79.62	65.59	81.65
	(− 6.99)	(− 5.14)	(− 8.91)	(− 7.89)	(− 8.44)	(− 9.29)	(− 11.88)
BioBERT-MTM	85.36	78.38	87.46	89.9	88.36	75.33	93.56
	(+ 0.74)	(+ 0.97)	(+ 0.57)	(+ 0.72)	(+ 0.30)	(+ 0.45)	(+ 0.12)
BioBERT-CFA-MTM	85.65	78.67	87.76	90.09	88.87	75.98	94.26
	(+ 1.03)	(+ 1.26)	(+ 0.87)	(+ 0.91)	(+ 0.81)	(+ 1.10)	(+ 0.82)

### Comparative analysis with previous studies

In this section, we compare the results of the final model BioBERT-CFA-MTM, which utilizes the proposed CFA module and fully-shared MTL method, with those of previous corresponding publications in the multi-task learning BioNER task. The results (F1 scores) on the same datasets are summarized in Table 4. Overall, our model outperforms previous studies in the BioNER task and achieves the best performance on all benchmark datasets. There are some valuable observations. First, the approaches based on the pre-trained language model, such as Akdemir et al. [33], Khan et al. [32], Tong et al. [40] and Chai et al. [34] generally outperform those based on the CNN and BiLSTM model, such as Crichton et al. [14], Wang et al. [28], Wang et al. [29], Yoon et al. [30] and Zuo et al. [31] This shows the power of using the pre-trained model as the encoder. Second, although the models of Akdemir et al. and Khan et al. are also based on the pre-trained BioBERT, the proposed BioBERT-CFA-MTM yields better performance because we combine the syntactic information and MTL method into BioBERT. Third, compared with the latest state-of-the-art model from Chai et al., BioBERT-CFA-MTM achieves substantial improvements on several datasets. This is because the former approach is based on the pre-trained XLNet model which is inferior to the biomedical domain-specific pre-trained BioBERT model. It also divides the parameters of the XLNet-CRF model into shared layers and task-specific layers while we share all the parameters of BioBERT-CFA-MTM across different datasets to learn more information from datasets.

## Discussion

### The effect of dimensions

**Fig. 6:**
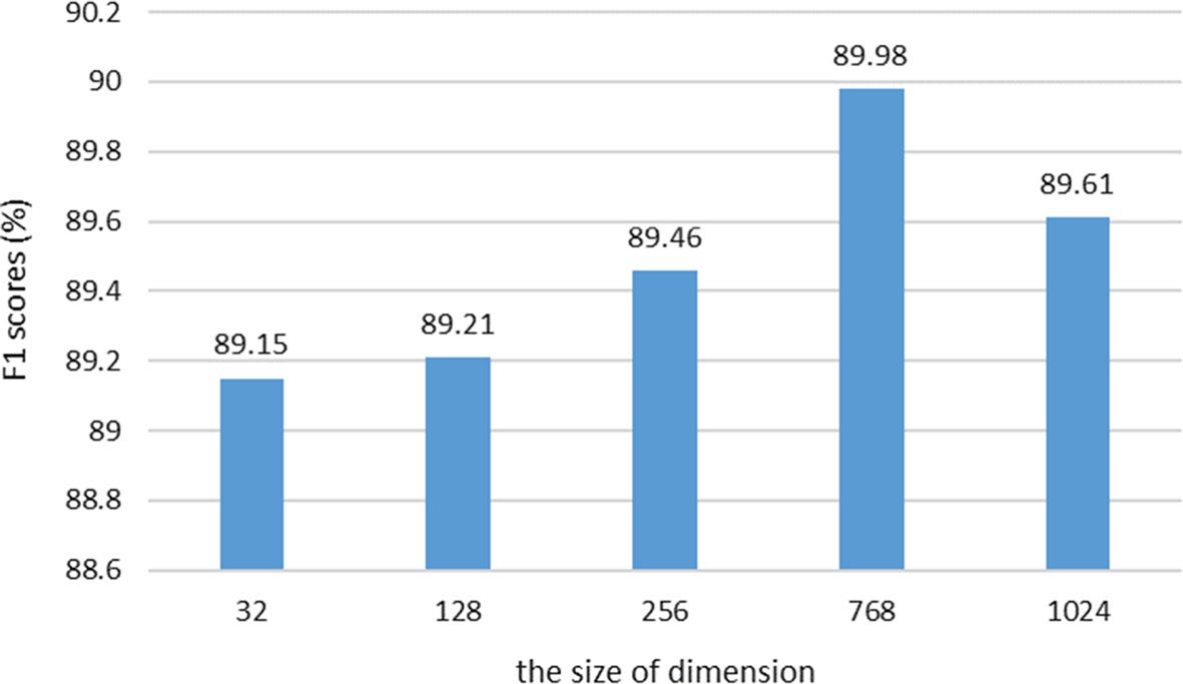
Impact of different sizes of dimension ${{d}_{2}}$

We analyze the influence of different initial dimension sizes for the syntactic label embedding in the CFA module. The syntactic labels are used for integrating with the context features into the syntactic features, which are introduced in Equation 4. Therefore, the dimension ${{d}_{2}}$ of the syntactic label embedding can affect the model performance. We test the different sizes of ${{d}_{2}}$ by 32, 128, 256, 768, 1024 in the BioBERT-CFA model with the features of POS labels on the NCBI-disease dataset. Figure 6 presents the results where the best performance is achieved at 768. Since the size of the vocabulary for potential syntactic labels is relatively small (less than 100), we assumed a small size of ${{d}_{2}}$ would achieve better F1 scores. But the result shows the least size 32 gets the worst performance. It can be interpreted that the dimension ${{d}_{2}}$ of the syntactic label embedding is much smaller than the dimension ${{d}_{1}}$ of the context feature embedding, which leads to lower weights for the syntactic labels in the CFA module. Contrarily, the size 768 is equal to the dimension ${{d}_{1}}$ and therefore achieves the best performance.

**Table 4:** Comparison of the proposed model with previous multi-task model

Model	BC2GM	JNLPBA	BC5CDR-Disease	NCBI-disease	Linnaeus	Species-800	BC5CDR-Chemical
Crichton et al. [14]	73.17	70.09	–	80.37	84.04	–	83.9
Wang et al. [28]	80.74	73.52	–	86.14	–	–	88.78
Wang et al. [29]	84.41	–	–	86.50	82.4	–	–
Yoon et al. [30]	79.73	78.58	84.08	86.36	–	–	93.31
Zuo et al. [31]	82.09	74.22	–	87.45	–	–	89.19
Akdemir et al. [33]	82.99	77.88	84.86	88.09	87.03	75.62	92.58
Khan et al. [32]	83.01	72.89	–	86.68	–	–	89.5
Chai et al. [34]	82.92	78.32	87.28	89.25	86.37	–	93.83
BioBERT-CFA-MTM	**85.65**	**78.67**	**87.76**	**90.09**	**88.87**	**75.98**	**94.26**

### The effect of the tag pair

In the proposed fully-shared MTL method, each input word sequence has attached a pair of tag identifiers, “$<tag>$” and “$</tag>$,” at the beginning and end of the sequence respectively. To prove the effectiveness of the strategy, we conduct experiments and show the results in Table 5. The BioBERT-MTM model is the fully shared MTL model which uses a pair of tag identifiers to distinguish between different datasets and achieve outstanding performance on the seven BioNER datasets. Then we remove the end tag “$</tag>$” and only keep the beginning tag “$<tag>$” for each input sentence. From the results of “w/o the end tag” model, removing the end tag strategy leads to a slight decline in the F1-scores. It shows that using a pair of tag identifiers positively affects the hidden representations of each word in the sentence more than using a single tag. If we remove the entire pair of tag identifiers, “$<tag>$” and “$</tag>$,” and only input the original sentence as the “w/o the tag pair” model, it degrades to the aforementioned baseline BioBERT-DM model. This method seriously damages the performance because it treats the sentences from different datasets as from the same source and does not distinguish between different datasets.

**Table 5:** The results of different fully shared MTL methods

Model	BC2GM	JNLPBA	BC5CDR-Disease	NCBI-disease	Linnaeus	Species-800	BC5CDR-Chemical
BioBERT-MTM	85.36	78.38	87.46	89.9	88.36	75.33	93.56
w/o the end tag	84.98	77.95	87.28	89.44	88.25	75.13	93.51
	(− 0.38)	(− 0.43)	(− 0.18)	(− 0.46)	(− 0.11)	(− 0.20)	(− 0.05)
w/o the tag pair	77.63	72.27	77.98	81.29	79.62	65.59	81.65
	(− 7.73)	(− 6.12)	(− 9.48)	(− 8.61)	(− 8.74)	(− 9.74)	(− 11.91)

### Analysis for datasets under the same type

To analyze the effect of entity types of the BioNER dataset, we train the BioBERT-DM and BioBERT-MTM model on the only two datasets of the disease type, i.e. NCBI-disease and BC5CDR-Disease, and show the results in Table 6. The “BioBERT-MTM (all)” denotes the aforementioned result of the BioBERT-MTM model where we train it on all seven datasets including other entity types. The BioBERT-DM model, where we directly mix the two disease datasets for training, does not gain satisfactory results compared with the single-task model BioBERT-STM. It shows that there are still some differences between the datasets of the same type and directly mixing them will degrade the performance. In contrast, BioBERT-MTM obtains good results, which proves again that the proposed fully-shared MTL method learns useful information across different datasets, even though these datasets are of the same type. Besides, the results of the BioBERT-MTM model trained on all datasets of multiple entity types are better than that trained on the two datasets of a single type, which shows that cross-type information in the biomedical domain improves the performance by using our MTL method.

**Table 6:** The results of using the datasets under the same type

Model	BC5CDR-Disease	NCBI-disease
BioBERT-STM	86.89	89.18
BioBERT-DM	86.66	89.35
	(− 0.23)	(+ 0.17)
BioBERT-MTM	87.13	89.55
	(+ 0.24)	(+ 0.37)
BioBERT-MTM (all)	87.46	89.9
	(+ 0.57)	(+ 0.72)

### Analysis for using the tag of type name

In the above experiments of BioBERT-MTM, where the tag pair is attached to the input sentence, we use the name of the dataset containing this sentence as the tag. Similarly, we can use the name of the corresponding entity type as the tag. For example, when the input sentence is from the Linnaeus dataset, the pair of tags will be “$< Species>$” and “$</Species>$.” As shown in Table 7, “TN” denotes the method using the name of the type and “DN” denotes the method using the name of the dataset. Bold marks the highest score among all methods. We found that the results of “DN” vastly outperform “TN” in most cases, which shows some differences exist in the datasets even of the same type. In addition, we combine the method “TN” and “DN” by attaching two pairs of tag identifiers at the beginning and end of the sentence respectively and name it “TN+DN.” The results of “TN+DN” are worse than “DN.” It shows that in the “TN+DN” method, too many tags are attached to the input sentence, and the model cannot understand the meaning of each tag well.

**Table 7:** Results of different tag methods

Model	Gene/protein		Disease		Species		Chemical
	BC2GM	JNLPBA	BC5CDR-Disease	NCBI-disease	Linnaeus	Species-800	BC5CDR-Chemical
BioBERT-STM	84.62	77.41	86.89	89.18	88.06	74.88	93.44
BioBERT-MTM							
TN	84.96	78.04	87.28	89.83	87.79	74.75	**93.64**
DN	**85.36**	**78.38**	**87.46**	**89.90**	**88.36**	**75.33**	93.56
TN+DN	85.03	78.32	87.23	89.65	88.12	75.14	93.25

### Case study

To better illustrate how our approach improves biomedical named entity recognition, we conduct a case study and list some practical prediction cases of the baseline single-task model and the two proposed multi-task models on several benchmark datasets. The examples are shown in Table 8, where true labels and predicted labels are underlined in the sentence for each model. In case 1, we need to recognize entities about the gene or protein type. BioBERT-MTM correctly detects the boundaries of gene entity “human Elk1 gene” compared with the baseline, possibly because the multi-task model could learn similar context expressions from other related datasets. BioBERT-CFA-MTM correctly detects the boundaries of gene entity “pseudogene Elk2,” while BioBERT-STM and BioBERT-MTM only detect the result “Elk2.” This can be interpreted that BioBERT-CFA-MTM learns the relations between “pseudogene” and “Elk2” from the corresponding lexical structures. In case 2, all models have correct prediction for the disease entity “cancer,” but only BioBERT-CFA-MTM correctly recognizes “inherited colorectal polyposis,” which is probably due to the effect of the CFA module. In case 3, BioBERT-STM fails to predict the species entity “Colwellia psychrerythraea 34H” while BioBERT-MTM is able to correctly detect it. Besides, “Alteromonadales” is recognized as a species entity in BioBERT-STM and BioBERT-MTM, however, this word is not an entity in the standard answer. In summary, BioBERT-CFA-MTM improves BioNER effectively because it learns more lexical structures from the syntactic information and shares useful information between multiple datasets by the fully-shared MTL method. Nevertheless, there are still some difficult cases that cannot be solved by our models. In case 4, the phrase “skin photosensitivity” is inferred as the disease entity by three models, but it is not an entity. The word “photosensitivity” is rare and it does not appear in the training set, and the expression of the phrase “skin photosensitivity” is similar to other skin diseases, e.g., “skin fragility syndrome” and “skin track,” therefore it is error-prone and hard to correctly recognize. Likewise, in case 5, because the species entity “rhubarb” is a rare word and it is difficult to identify according to the context, our models fail to recognize it.

**Table 8:** Case study of the prediction examples from different models

Dataset: BC2GM	Dataset: BC2GM entity type: gene/protein	
Case 1	True label	Structural organization of the human Elk1 gene and its processed pseudogene Elk2.
	BioBERT-STM	Structural organization of the human Elk1 gene and its processed pseudogene Elk2.
	BioBERT-MTM	Structural organization of the human Elk1 gene and its processed pseudogene Elk2.
	BioBERT-CFA-MTM	Structural organization of the human Elk1 gene and its processed pseudogene Elk2.
Dataset: NCBI-disease entity type: disease	Entity type: disease	
case 2	True label	Inherited colorectal polyposis and cancer risk of the APC I1307K polymorphism.
	BioBERT-STM	Inherited colorectal polyposis and cancer risk of the APC I1307K polymorphism.
	BioBERT-MTM	Inherited colorectal polyposis and cancer risk of the APC I1307K polymorphism.
	BioBERT-CFA-MTM	Inherited colorectal polyposis and cancer risk of the APC I1307K polymorphism.
Dataset: Linnaeus	entity type: species	
Case 3	True label	Other species represented are Colwellia psychrerythraea 34H and Shewanella oneidensis, that belong to the Alteromonadales family.
	BioBERT-STM	Other species represented are Colwellia psychrerythraea 34H and Shewanella oneidensis, that belong to the Alteromonadales family.
	BioBERT-MTM	Other species represented are Colwellia psychrerythraea 34H and Shewanella oneidensis, that belong to the Alteromonadales family.
	BioBERT-CFA-MTM	Other species represented are Colwellia psychrerythraea 34H and Shewanella oneidensis, that belong to the Alteromonadales family.
Dataset: NCBI-disease	Entity type: disease	
case 4	True label	Skin photosensitivity may also be present.
	BioBERT-STM	Skin photosensitivity may also be present.
	BioBERT-MTM	Skin photosensitivity may also be present.
	BioBERT-CFA-MTM	Skin photosensitivity may also be present.
Dataset: Linnaeus	Entity type: species	
case 5	True label	Scanagati then recommended the continuation of the electuary made of emollient, guaiacum resin, balsam, rhubarb, and nitre.
	BioBERT-STM	Scanagati then recommended the continuation of the electuary made of emollient, guaiacum resin, balsam, rhubarb, and nitre.
	BioBERT-MTM	Scanagati then recommended the continuation of the electuary made of emollient, guaiacum resin, balsam, rhubarb, and nitre.
	BioBERT-CFA-MTM	Scanagati then recommended the continuation of the electuary made of emollient, guaiacum resin, balsam, rhubarb, and nitre.

## Conclusions

In this paper, we propose a novel fully-shared multi-task learning model based on the pre-trained BioBERT with a new attention module to integrate the auto-processed syntactic information for the BioNER task. The proposed attention module CFA extracts appropriate features from syntactic information and weights these features to enhance BioNER. The proposed multi-task learning method shares all parameters to capture useful information from different datasets. We conducted a large number of experiments on seven benchmark BioNER datasets and our methods achieved the best results on all datasets. The experiment results and case studies demonstrate the importance of the proposed CFA module and fully shared MTL method used in our model. In the future, we expect to employ biomedical-specific syntactic toolkits instead of the general-purpose toolkit to further improve the performance for CFA, and apply the proposed approach to other sequence tagging tasks.

## Data Availability

The datasets and code are available at are available at https://github.com/zzy1026/BioBERT-CFA-MTM. BioBERT pre-training parameters were provided by https://github.com/dmis-lab/biobert-pytorch.

## Abbreviations

BioNER: Biomedical named entity recognition
CFA: Combined feature attention
MTL: Multi-task learning
POS: Part-of-speech
CRF: Conditional random field
CNN: Convolutional neural network
BiLSTM: Bidirectional long short-term memory
BERT: Bidirectional encoder representations from transformers
SCT: Stanford CoreNLP Toolkit

## Footnotes

1 https://github.com/cambridgeltl/MTL-Bioinformatics-2016.

2 https://github.com/dmis-lab/biobert-pytorch.

3 http://pytorch.org/.

4 https://github.com/chakki-works/seqeval.
